# Deep-learning-based real-time prediction of acute kidney injury outperforms human predictive performance

**DOI:** 10.1038/s41746-020-00346-8

**Published:** 2020-10-26

**Authors:** Nina Rank, Boris Pfahringer, Jörg Kempfert, Christof Stamm, Titus Kühne, Felix Schoenrath, Volkmar Falk, Carsten Eickhoff, Alexander Meyer

**Affiliations:** 1 Department of Cardiothoracic and Vascular Surgery, German Heart Center Berlin, Augustenburger Platz 1, 13353 Berlin, Germany; 2 DZHK (German Centre for Cardiovascular Research), Partner Site Berlin, P.O. Box 65 21 33, 13316 Berlin, Germany; 3 Institute for Computer-assisted Cardiovascular Medicine, Charité–Universitätsmedizin Berlin, Augustenburger Platz 1, 13353 Berlin, Germany; 4 Berlin Institute of Health, Anna-Louisa-Karsch-Str. 2, 10178 Berlin, Germany; 5 Department of Cardiothoracic Surgery, Charité – Universitätsmedizin Berlin, Charitéplatz 1, 10117 Berlin, Germany; 6 Department of Health Sciences and Technology, ETH Zürich, Leopold-Ruzicka-Weg 4, 8093 Zürich, Switzerland; 7 Center for Biomedical Informatics, Brown University, 233 Richmond Street, Providence, RI 02912 USA

**Keywords:** Diagnosis, Preventive medicine

## Abstract

Acute kidney injury (AKI) is a major complication after cardiothoracic surgery. Early prediction of AKI could prompt preventive measures, but is challenging in the clinical routine. One important reason is that the amount of postoperative data is too massive and too high-dimensional to be effectively processed by the human operator. We therefore sought to develop a deep-learning-based algorithm that is able to predict postoperative AKI prior to the onset of symptoms and complications. Based on 96 routinely collected parameters we built a recurrent neural network (RNN) for real-time prediction of AKI after cardiothoracic surgery. From the data of 15,564 admissions we constructed a balanced training set (2224 admissions) for the development of the RNN. The model was then evaluated on an independent test set (350 admissions) and yielded an area under curve (AUC) (95% confidence interval) of 0.893 (0.862–0.924). We compared the performance of our model against that of experienced clinicians. The RNN significantly outperformed clinicians (AUC = 0.901 vs. 0.745, *p* < 0.001) and was overall well calibrated. This was not the case for the physicians, who systematically underestimated the risk (*p* < 0.001). In conclusion, the RNN was superior to physicians in the prediction of AKI after cardiothoracic surgery. It could potentially be integrated into hospitals’ electronic health records for real-time patient monitoring and may help to detect early AKI and hence modify the treatment in perioperative care.

## Introduction

Acute kidney injury (AKI) is a major postoperative complication after cardiothoracic surgery. It is an independent risk factor for early and long-term mortality^1–4^ and is strongly associated with increased hospital costs and length of stay^5–7^.

AKI is defined as a major increase of serum creatinine or a strong decline in urine output^8^. Compromised renal blood flow and cardiopulmonary bypass play a critical role in the development of AKI, but overall its etiology is highly multifactorial^9–12^.

Early detection of patients at high risk of developing AKI allows for early therapeutic intervention prior to the onset of anuria and its complications such as acidosis, hyperkalemia, or volume overload as well as long-term complications such as lung injury, sepsis and chronic kidney disease^13–16^. In a pilot study in 2011 it was demonstrated that in patients with AKI stage I, early nephrologist consultation can avert progression to higher AKI stages^17^. It was also shown that delayed nephrologist involvement (48 h after AKI onset) in critically ill patients was associated with an increase of mortality and dependence on dialysis^18^. An immediate post-operative “KDIGO care bundle” (optimization of volume status and hemodynamics, avoidance of nephrotoxic drugs and hyperglycemia) in high-risk patients has been shown to reduce cardiac surgery-associated AKI^19^.

Although several classical clinical risk scores for the prediction of postoperative AKI exist, none of them is specifically recommended by guidelines^20–26^. With few exceptions they rely on patient demographics, disease history and the type of surgery and require time-consuming manual data collection and calculation. Furthermore, they are usually based on static properties or single point-in-time measurements that cannot adapt to the often rapid and dramatic changes that occur in the postoperative setting.

Increased digitization of medical information opens up new alternatives for early prediction of postoperative complications that might potentially be integrated into existing electronic health record (EHR) software. A vast amount of data with high temporal resolution is collected during a hospital stay. Effectively processing such high-dimensional data in a parallelized way, however, goes far beyond the capabilities of the human brain^27^. Machine learning (ML) offers a potential solution to this problem.

Previous studies investigating the performance of ML models in predicting AKI have yielded promising results^28–35^. However, studies directly comparing the predictive performance of ML models against experienced physicians in the prediction of postoperative AKI on time-series data of real clinical cases are highly needed.

We therefore developed a recurrent neural network (RNN) that allows real-time predictions of AKI within the first 7 postoperative days following cardiothoracic surgery based on routinely collected variables (features). This model was then compared to the performance of experienced health-care professionals.

## Results

### Performance of the RNN based prediction

A complete description of the study population, patient selection process, development of the ML model, and the experimental design of our RNN-vs-human comparison can be found in the ‘Methods’ section.

In summary, we retrospectively analysed EHR time series data with high temporal resolution (up to 1 min) generated at a tertiary care center for cardiovascular diseases. Based on *n* = 2224 admissions, we developed an RNN that continuously (every 15 min) predicted the probability of developing AKI defined as KDIGO^8^ stage 2 or 3 within the first 7 days after cardiothoracic surgery.

Supplementary Tables 1–4 show a comparison of baseline characteristics between AKI- and non-AKI cases in the training, balanced and imbalanced test set and the whole study population before matching AKI- and non-AKI cases.

Table 1 shows the performance metrics of our RNN evaluated on an independent test set with *n* = 350 patients. The model achieved an area under curve (AUC) (95% confidence interval (CI)) of 0.893 (0.862–0.924). In addition, we trained a model with only serum creatinine as input and yielded an AUC of 0.805 (0.768–0.842). Thus, the addition of further parameters led to an absolute increase of around 10 percentage points in the AUC. However, a model using all features but creatinine and glomerular filtration rate (GFR) (the GFR is calculated from creatinine) performed almost as good as the full model with an AUC of 0.887 (0.855–0.919)—probably due to high correlation between creatinine and other features, e.g., urea. For further performance metrics of these reduced models see Supplementary Tables 5 and 6.

**Table 1:** Model performance metrics for balanced test set.

Threshold-independent metrics, (95% CI)			Threshold-dependent metrics, (95% CI)						
AUC	PR_AUC	$\overline {{{\mathbf{MSE}}}} _{{{\mathbf{pat}}}}$	Acc	Sens	Spec	F1	FPR	NPV	PPV
0.893 (0.862–0.924)	0.903 (0.873–0.933)	0.124 (0.090–0.159)	0.825 (0.786–0.863)	0.853 (0.802–0.904)	0.798 (0.741–0.855)	0.826 (0.776–0.876)	0.202 (0.145–0.259)	0.851 (0.799–0.903)	0.801 (0.745–0.857)

*n* = 350 admissions/patients.

*AUC* area under curve, *PR_AUC* precision-recall AUC, $\overline {{\mathrm{MSE}}} _{\mathrm{pat}}$ mean of the brier score of each patient, *Acc* accuracy, *Sens* sensitivity, *Spec* specificity, *F1* F_1_-score, *FPR* false-positive rate, *NPV* negative predictive value, *PPV* positive predictive value, *CI* confidence interval. The threshold for positive/negative class prediction was set to 0.41, leading to a sensitivity of 0.850 on cross-validation folds in the training set.

A table with the model performance metrics derived from an imbalanced test set with incidence rate of 10% AKI (see Supplementary Results 1) can be found in Supplementary Table 7. In addition, we analysed some examples of the predictions of individual patients including false-positive and false-negative predictions. These can be found in Supplementary Figs. 1–3.

### RNN vs. human-level performance—experimental design

**Fig. 1:**
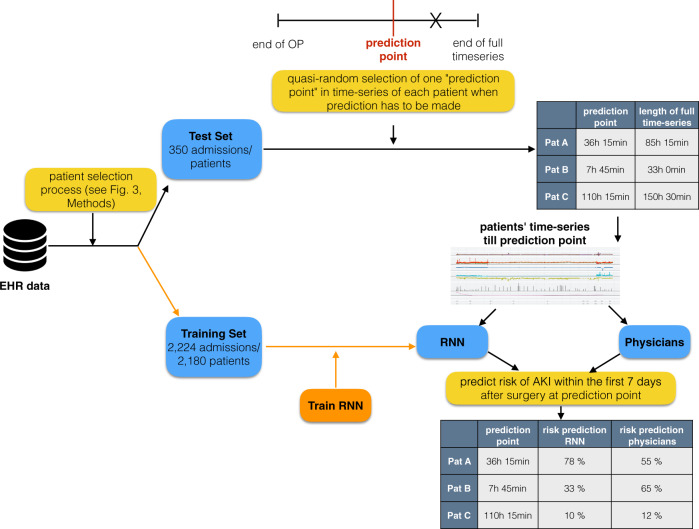
Experimental design for performance comparison of recurrent neural network (RNN) against physicians. The electronic health record (EHR) data was split into a training and a test set. The training set was used for the development of the RNN (orange path). For each patient (Pat) in the test set, a quasi-random ‘prediction point’ in the time-series was chosen (for more information about quasi-randomness see ‘Methods’). EHR data up to this prediction point was given to physicians and RNN (the rest of the time series data, here denoted as X, was hidden). Both physicians and RNN, had to make a prediction for postoperative AKI at this prediction point.

We set up an experiment to compare our ML model against experienced physicians (Fig. 1). For each of the *n* = 350 patients of our balanced test set a quasi-random point in time in their observation period was chosen, further denoted as ‘prediction point’ (For more information about quasi-random samples see the ‘Methods’ section.).

At the chosen prediction point, seven experienced physicians and the ML model each had to make a prediction (between 0 and 100%) of how likely the patient was to develop AKI within the first 7 days after surgery.

All time series information up to the ‘prediction point’ was graphically displayed for the physicians to mimic the electronic patient chart.

### Performance of RNN and physicians

The performance of our RNN and the physicians’ assessment can be found in Table 2 (Note that the metrics of the RNN are slightly different from those in section ‘Performance of the RNN based prediction’. The reason is that in the RNN vs. human experiment only one prediction point per patient was evaluated, whereas for the complete evaluation of the RNN all predictions of the whole observation periods for all patients were evaluated.).

**Table 2:** Performance metrics of recurrent neural network (RNN) and physicians on a balanced test set.

	Threshold-independent metrics, (95% CI)			Metrics based on a threshold of 0.5 for positive/negative classification, (95% CI)						
	AUC	PR_AUC	Brier	Acc	Sens	Spec	F1	FPR	NPV	PPV
RNN	0.901 (0.870–0.932)	0.907 (0.877–0.937)	0.122 (0.088–0.156)	0.846 (0.808–0.884)	0.851 (0.798–0.904)	0.840 (0.787–0.894)	0.847 (0.797–0.897)	0.160 (0.106–0.214)	0.850 (0.797–0.903)	0.842 (0.788–0.896)
Physicians	0.745 (0.699–0.791)	0.747 (0.701–0.793)	0.217 (0.174–0.260)	0.711 (0.664–0.759)	0.594 (0.521–0.667)	0.829 (0.773–0.884)	0.673 (0.609–0.738)	0.171 (0.116–0.227)	0.671 (0.601–0.741)	0.776 (0.715–0.838)

*n* = 350 admissions/patients.

*AUC* area under curve, *PR_AUC* precision-recall AUC, *Brier* Brier score, *Acc* accuracy, *Sens* sensitivity, *Spec* specificity, *F1* F_1_-score, *FPR* false-positive rate, *NPV* negative predictive value, *PPV* positive predictive value, *CI* confidence interval.

The median (interquartile range (IQR)) prediction value for the physicians was 0.36 (0.15–0.70) vs. 0.51 (0.12–0.86) for the RNN.

**Fig. 2:**
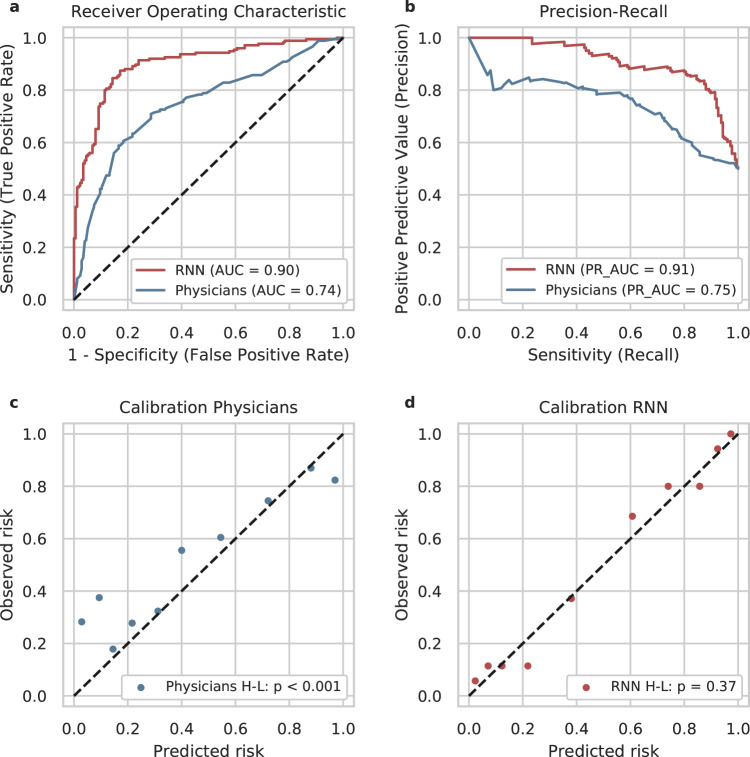
Discrimination and calibration of the predictions of recurrent neural network (RNN) and physicians. **a** receiver operating characteristics (ROC), **b** precision-recall curve**, c** calibration of physicians**, d** calibration of RNN. AUC area under curve. H-L Hosmer-Lemeshow-Test^36^, PR_AUC precision-recall AUC. The RNN outperformed clinical physicians regarding AUC (**a**) and PR_AUC (**b**). Physicians systematically underestimated the risk of acute kidney injury (predicted risks < observed risks, **c**). In contrast, the RNN was overall well calibrated (**d**).

Across all metrics, the RNN outperformed the physicians. We obtained an AUC of 0.901 for the RNN vs. 0.745 for the physicians (*p* < 0.001, *Z* = 6.85, DeLong’s test). The receiver operating characteristic (ROC) curves and the precision-recall curves are displayed in Fig. 2a and Fig. 2b, respectively.

The mean of our predictive quality score S (S = r, if the patient developed AKI and S = 1−r, if the patient did not develop AKI) was significantly higher for the RNN than for the experienced physicians (0.754 vs 0.639, *p* < 0.001, *t*-statistic = 8.47, df = 349, paired *t*-test).

In addition, we investigated the calibration of the RNN’s and physicians’ predictions. Calibration describes how close the predicted probabilities are to the observed frequencies. A perfectly calibrated model would have one point at (0,0) and one at (1,1) in a calibration plot (it would always predict 0 for negatives and 1 for positives). For a well-calibrated model, the points lie on the diagonal between (0,0) and (1,1). Figure 2c illustrates that in the intervals of high prediction values of physicians, the predicted frequencies of AKI largely correspond to the observed frequencies (upper right part of the calibration curve). However, for several patients that developed AKI, physicians predicted low AKI probabilities (false-negative predictions, lower left part of the calibration curve). This is also reflected in the observation that the physicians’ median (IQR) prediction value was lower than the RNN’s (Physicians: 0.36 (0.15–0.70) and RNN: 0.51 (0.12–0.86)). Overall the physicians’ predictions were not well calibrated (*p* < 0.001, *Χ*^2^ = 165.5, df = 8, Hosmer-Lemeshow-test^36^).

In contrast, Fig. 2d displays a very well calibration (*p* = 0.37, *Χ*^2^ = 8.67, df = 8, Hosmer-Lemeshow-test) for the RNN, with most of the points lying very close to the diagonal, even in intervals of low prediction values.

We investigated the performance of our RNN and physicians at different points in time before the event (AKI or non-AKI/discharge) (see Table 3). Not-surprisingly, both, humans and RNN, performed worse when the event was further away in time. However, low sensitivity rates could also be observed when the event was very close (≤2 h). In this group the median total observation length was very short, meaning that patients who developed AKI, developed it rapidly after surgery. Thus, there was probably not enough information available before the event to reliably predict AKI. However, even in this interval, the RNN reached a sensitivity of 0.789.

**Table 3:** Performance metrics of recurrent neural network (RNN) and physicians in temporal dependence to the event.

Predictor	Time to event	patients	AKI	MOL	AUC	PR_AUC	Brier	Acc	Sens	Spec	F_1_	FPR	NPV	PPV
RNN	0 h to 2 h	54	19	8.3 h	0.913	0.837	0.113	0.870	0.789	0.914	0.811	0.086	0.889	0.833
Physicians	0 h to 2 h	54	19	8.3 h	0.709	0.552	0.199	0.759	0.632	0.829	0.649	0.171	0.806	0.667
RNN	2 h to 6 h	63	29	12.5 h	0.881	0.88	0.13	0.825	0.862	0.794	0.820	0.206	0.871	0.781
Physicians	2 h to 6 h	63	29	12.5 h	0.853	0.861	0.152	0.794	0.793	0.794	0.780	0.206	0.818	0.767
RNN	6 h to 12 h	63	34	17.8 h	0.942	0.948	0.088	0.921	0.971	0.862	0.930	0.138	0.962	0.892
Physicians	6 h to 12 h	63	34	17.8 h	0.811	0.798	0.19	0.746	0.618	0.897	0.724	0.103	0.667	0.875
RNN	12 h to 24 h	74	42	36.4 h	0.888	0.921	0.128	0.824	0.881	0.750	0.851	0.250	0.828	0.822
Physicians	12 h to 24 h	74	42	36.4 h	0.693	0.706	0.257	0.689	0.667	0.719	0.709	0.281	0.622	0.757
RNN	24 h to 48 h	60	31	46.4 h	0.890	0.899	0.142	0.817	0.774	0.862	0.814	0.138	0.781	0.857
Physicians	24 h to 48 h	60	31	46.4 h	0.718	0.774	0.246	0.633	0.387	0.897	0.522	0.103	0.578	0.800
RNN	48 h to 168 h	36	20	99.0 h	0.875	0.929	0.132	0.806	0.750	0.875	0.811	0.125	0.737	0.882
Physicians	48 h to 168 h	36	20	99.0 h	0.647	0.741	0.274	0.611	0.400	0.875	0.533	0.125	0.538	0.800

*AKI* number of patients with acute kidney injury, *MOL* median total observation length, *AUC* area under curve, *PR_AUC* precision-recall AUC, *Brier* Brier score, *Acc* accuracy, *Sens* sensitivity, *Spec* specificity, *F1* F_1_-score, *FPR* false-positive rate, *NPV* negative predictive value, *PPV* positive predictive value.

## Discussion

We developed an RNN for real-time prediction of postoperative AKI within 7 days after cardiothoracic surgery—based on routinely collected features during the hospital stay and then retrospectively validated it on an independent test set.

To test the clinical significance, we performed a side-by-side comparison of our model against experienced physicians. Such direct comparisons are highly needed, but hardly ever performed in clinical ML studies. We had expected our model to perform nearly as well as the physicians, and had designed our study as a non-inferiority-experiment. Surprisingly, our RNN significantly outperformed experienced clinicians in terms of the mean of our performance metric S. (S indicates how close a prediction is to the observed outcome). In addition, the model reached a significantly higher AUC than the physicians (0.901 vs. 0.745, *p* < 0.001, DeLong’s test) and was overall well calibrated (Hosmer-Lemeshow-Test: *p* = 0.37 vs. *p* < 0.001 for physicians).

Physicians showed an overall low sensitivity of 0.594 at AKI prediction. They predicted lower risk probabilities in general. They reached a maximum sensitivity of 0.793 for the 2–6 h interval before the event and a minimum sensitivity of 0.387 for the 24–48 h interval before the event. Thus, they systematically underestimated the risk of AKI. This suggests that physicians mainly recognize AKI stage 3 or dialysis and that lower AKI stages are erroneously considered unproblematic. It has been demonstrated, however, that even minor increases in serum creatinine after cardiac surgery are associated with an increased mortality risk^37^.

The participating physicians each had at least one year working experience on a cardiothoracic intensive care unit (ICU), but were no specialists in nephrology. This reflects a realistic clinical setting on an ICU, where nephrologists are usually not available around the clock.

In contrast to the physicians, our RNN yielded an overall high sensitivity of 0.851 with a maximum sensitivity of 0.971 in the 2–6 h interval before the event and a minimum sensitivity of even 0.750 in the 48–168 h interval before the event. In summary, our RNN was superior to experienced physicians in the prediction of AKI after cardiothoracic surgery.

From a modeling point of view, our RNN could easily be integrated into an EHR system. It does not require any additional human input as all data transformation is implemented programmatically. Allowing for personalized predictions, it may enable earlier identification and intervention in high-risk patients and thus contribute to an improvement of patient care and safety. However, the transfer of such a retrospective model from research to real implementation raises additional challenges. Technical barriers, data security when exporting personal data to external software systems, and business considerations may be diverse and can conflict with each other.

Our model achieved highly accurate results with an overall AUC of 0.893 in our internal validation. It outperformed existing classical prediction models that are based on logistic regression from static pre- and intraoperative variables, as well as a dynamic model that predicted AKI at three points in time (pre-operative, at ICU admittance and 24 h after ICU admittance). These models reached AUCs ranging from 0.72–0.85 in their respective internal validation cohorts and used slightly different definitions of AKI^20–26,38^ (see Table 4). The proposed model does not create additional workload for physicians, as it only used routinely collected data of the EHR. As such, it only employs data that is available at the time of prediction and all data transformations are implemented programmatically. It is worth noting that the model performed very well, although it was built on a relatively small sample size of 2224 admissions.

**Table 4:** Comparison between classical prediction models^20^ based on logistic regression and our recurrent neural network (RNN).

Authors, model	Sample size derivation	Sample size internal validation	Validation method	“Real-time” prediction	Predicted outcome	Manual calculation	AUC on internal validation
Chertow et al., CICSS^21^	42,773	42,773	100-sample bootstrap	No	30 days post-op. AKI	Yes	0.76 (AUC on derivation cohort)
		3795	Prospective validation				Not reported
Brown et al., NNECDSG^38^	8363	8363	Bootstrap validated C-index (AUC)	No	Severe post-op. AKI (eGFR < 30 ml/min)	Yes	0.72* (0.68–0.75)
Palomba et al., AKICS^24^	603	215	Prospective validation	No	7 days post-op. AKI	Yes	0.85 (0.8–0.9)
Aronson et al., MCSPI^25^	2381	2420	Split sample validation	No	Renal dysfunction or renal failure (dialysis or evidence of renal failure at autopsy)	Yes	0.80
Wijeysundera et al., SRI^26^	10,751	10,751	200-sample bootstrap	No	Post-op. renal replacement therapy	Yes	0.81* (0.78–0.84)
		2566	Prospective validation				0.78 (0.72–0,84)
Mehta et al., STS (Mehta)^23^ simplified model	449,524	86,009	Independent sample	No	Post-op. dialysis	Yes	0.83
Thakar et al., Cleveland Clinic^22^	15,838	15,839	Split sample validation	No	Post-op. dialysis	Yes	0.82 (0.80–0.85)
Jiang et al., Dynamic Predictive Score^67^	6081	1152	Independent sample	No	AKI ≥ stage 1 KDIGO	Yes	0.74 preoperative, 0.75 at ICU admission, 0.82 postoperative
This study, RNN	2224	350	Independent Sample (balanced, incidence 50%)	Yes	7 days post-op. AKI stage 2 or 3	No	0.89 (0.86–0.92)
		1945	Independent sample (imbalanced, incidence 10%)				0.85 (0.83–0.86)

*AKI* acute kidney injury, *AUC* area under curve.

Previous studies have demonstrated the benefits of using ML for AKI prediction. Thottakkara et al.^28^ applied different ML approaches to forecast postoperative AKI and observed promising performances in their internal validation cohort (AUC between 0.797 and 0.858). Bihorac et al.^29^ used an ML algorithm to assess the risk of 8 postoperative complications including AKI and reported an AUC of 0.80 (0.79–0.80) for AKI prediction. The approach of both studies, however, relied exclusively on static, mostly preoperative features.

A multi-center ward-based AKI prediction model was developed by Koyner et al.^39^ using a discrete time survival model with an AUC (95% CI) of 0.76 (0.76–0.77) for AKI of at least stage 2.

In 2018, Koyner et al.^31^ published another study using EHR data for AKI risk prediction and reached an AUC (95% CI) of 0.90 (0.90–0.90) for predicting stage 2 AKI within the next 24 h and 0.87 (0.87–0.87) within the next 48 h. Cheng et al.^32^ built ML models to forecast AKI over various time horizons and obtained an AUC of 0.765 (prediction one day before the event). In these studies, however, the urine output criterion of AKI, a central component in the KDIGO definition was not integrated, which can lead to a false-negative classification of AKI cases. In our training and test cohort around 30% of the AKI cases were defined by the urine criteria of KDIGO (see Supplementary Table 8). We can assume that a substantial proportion of the patients in the above studies would also have met the urine criteria first. Probably not all of them have been classified as false-negative, as they might have met the creatinine criterion at a later stage. In our population, 11% of the AKI-cases in the training set and 12% in the test set exclusively fulfilled the urine criterion and would have been diagnosed false-negatively without this criterion. The median (IQR) diagnosis delay of patients who met both criteria within 7 postoperative days was 14.0 h (6.3–27.3 h) in the training set and 13.3 h (5.3–22.4 h) in the test set. Especially in models with short prediction horizons, there is a high risk that the prediction of imminent AKI and consequently initiation of preventive measurements is delayed when not integrating the urine criterion.

In addition, these previous models were restricted to patients with a serum creatinine of <3 mg/dl (Koyner et al.) or even normal serum creatinine level and a GFR of at least 60 ml/min/1.73 m^2^ (Cheng et al.) at admission.

Mohamadlou et al.^40^ developed an ML algorithm based on EHR data for detection of AKI at onset and prediction of AKI 12, 24, 48, and 72 h before onset. They reported AUCs from 0.872 (onset) to 0.728 (72 h before onset).

Another study for continuous AKI prediction on a large data set was performed by Tomašev et al.^34^. The developed RNN predicted AKI stage 2 or 3 with an AUC of 0.971 24 h before onset.

Also in these studies the urine output criterion of AKI was not incorporated. In addition, in the study of Tomašev et al. only patients were included for whom at least one year of EHR data were available before admission. They added aggregate features of up to five years of historical information of each individual patient. This approach requires that patients are already known in the admitting hospital, which is often not the case. It is unclear how their algorithm would perform on patients without any prior medical history. In contrast, we used a real uncurated data stream in our model that only contained information generated after admission.

Meyer et al.^35^ used an RNN to predict AKI requiring dialysis, mortality and postoperative bleeding after cardiac surgery using routinely collected parameters within the first 24 hours after surgery. The deep-learning model provided very accurate predictions (positive predictive value (PPV)/sensitivity for AKI: 0.87/0.94) that outperformed usual clinical risk scores.

Our model predicted AKI in a time frame up to 7 days after cardiothoracic surgery. Compared to the observation windows of the studies mentioned above, this is a much longer time period. Events in the near future are usually easier to predict than those in the more distant future. To intervene early when the kidneys are merely at risk of injury, a longer prediction window might be necessary. It has been shown that early intervention can prevent AKI or its progression to higher stages^17,19^. Therefore, the prediction of our model was not limited to AKI requiring dialysis, but included the prediction of AKI stages 2 or 3 according to the KDIGO definition.

To conclude, based on a relatively small sample size, we developed a highly accurate model for the prediction of AKI after cardiac surgery that significantly outperformed experienced physicians, could potentially be integrated into EHR systems and might prevent severe complications following AKI through real-time patient surveillance. In a long-term perspective, an extension of the application from a simple risk prediction model to treatment decision support tool is also conceivable.

This study has several shortcomings. The observation periods of the included patients varied widely in length. For most patients it ended in <3 days while some outliers lasted for up to 7 days. We only used the start of nephrotoxic drug administration as a feature. Consideration of exact dose, administration route (e.g., i.v., p.o, …), and administration length could reflect the underlying pharmacodynamics better and improve the prognostic performance.

Our RNN is currently cohort specific for cardiothoracic surgery patients that most likely have different characteristics and risk factors than, e.g., neurosurgical patients. Implementing the same approach on other patient cohorts could give a deeper insight into the generalizability of our method.

Our study is retrospective. Thus, in our RNN vs. physicians head-to-head comparison, physicians only received EHR data and could not clinically evaluate patients. Information such as volume status (except for weight), general condition, etc. or additional examinations (e.g., ultrasound) were not available to them and to the RNN. This deviation from the physicians’ usual workflow in clinical practice may explain some of the observed performance deficits. Real clinical data can be very noisy, leading to reduced performance and greater burden of deploying completely automated systems. This stresses once again the fact that artificial intelligence should be utilised in support systems for physicians and not as their replacement.

External validation trials should be performed on prospective data. In addition, they should focus on usage and acceptance of a system such as the one described here in a real clinical setting.

## Methods

### Ethics and reporting guideline

This study was approved by the institutional data protection officer and ethics committee of Charité – Universitätsmedizin Berlin (EA2/180/17). The approval included the collection of data on implied consent. We only used retrospective data and the patients were not actively involved in the study. The requirement of informed consent of the participating physicians was waived by the Institutional Review Board (IRB) of Charité – Universitätsmedizin Berlin due to anonymized data acquisition. Reporting of development and validation of the prediction model follows widely the guideline of the TRIPOD statement^41^.

### Patient selection process

We retrospectively analysed EHR time series data generated between October 2012 and February 2018 at a tertiary care center for cardiovascular diseases.

We included adult patients (18+) that were admitted at least once to the operating theatre for cardiothoracic surgery (15,564 admissions/13,895 patients). We excluded patients without any creatinine or urine flow values, patients receiving hemodialysis before the end of the operation or having a baseline creatinine level ≥4.0 mg/dl (2322 admissions/1487 patients).

Within this collection of 12,978 admissions, 1308 cases were identified with severe postoperative AKI defined as stage 2 or 3 according to KDIGO AKI guidelines—briefly, an increase in serum creatinine to at least twice the baseline value or a decrease in urine flow < 0.5 ml/kg/h for ≥12 h.

As AKI can develop over multiple days, we defined a study period of 7 days after cardiothoracic surgery. The global AKI label of a patient was set positive when the KDIGO criteria stage 2 or 3 was fulfilled at any point within these 7 postoperative days.

The observation time of each patient started when the patient was transferred to the ICU or recovery room. It ended when the patient was either discharged, or when the KDIGO criteria for AKI stage 2 or 3 were fulfilled, or after 7 days after the end of the first surgery.

Each AKI-case was assigned a control out of the non-AKI pool (11,670 admissions/11,046 patients). The controls were matched to the cases on observation length. Thus, we generated a balanced data set that we then randomly split into a training set (85%, 2224 admissions/2180 patients) and the remaining set (15%, 392 admissions/patients) while keeping the cases with their respective controls.

For the 392 patients of the remaining set we manually checked physicians’ notes in the EHR data and consequently excluded 28 patients. Exclusion criteria were primarily insufficient documentation of the type of surgery, false recording of surgery times or notion of end-stage kidney disease in the patients’ history that was not detected by automated filtering.

**Fig. 3:**
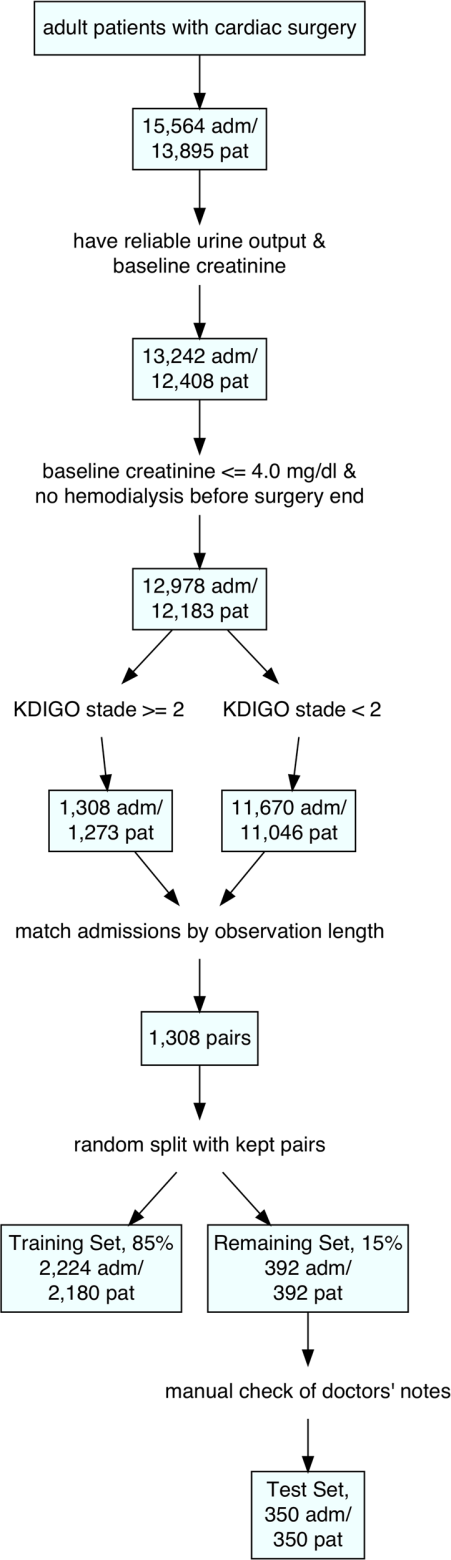
Flow chart of patient selection process. adm admissions, pat patients.

Out of this set, we randomly selected 350 patients that formed the final test set for model evaluation and comparison with human-level performance. A detailed flow chart of the patient selection process is shown in Fig. 3.

The baseline characteristics were well balanced between the training and the test and are summarized in Supplementary Table 8.

**Fig. 4:**
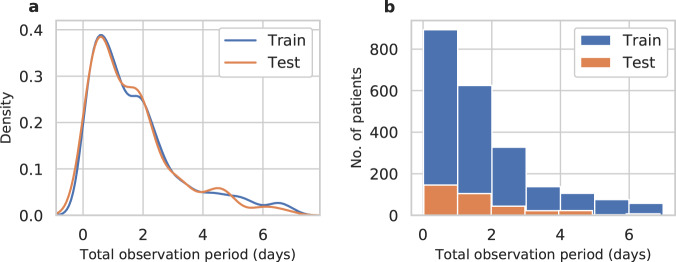
Total observation period for the training and test set. **a** Density distribution. **b** Histogram. For most patients the observation period ended within three days after surgery.

The density distribution and a histogram of the observation periods for patients in the training and test sets is shown in Fig. 4. Most patients were either discharged or diagnosed with AKI within the first 3 days after the first surgery.

### Feature selection and preprocessing

We developed our model based on 96 routinely collected clinical parameters. Table 5 gives an overview of all considered features. They can be grouped into static features (e.g., most patient and surgery characteristics, 25 features) that do not change over the observation period and frequently measured dynamic features (e.g., lab values, vital signs, blood gas values and fluid output, 49 features). In addition, we included a variety of widely administered agents that have been reported to potentially cause nephrotoxic effects^42–47^ (22 features).

**Table 5:** Input feature overview.

Feature Group (no. features)	Features
Patient characteristics (4)	Age, sex, weight, height
Laboratory results (25)	Phosphate, total bilirubin, baseline creatinine, creatinine, baseline urea, urea, glomerular filtration rate, creatine kinase (CK), CK-MB, red blood count, white blood count, platelets, C-reactive protein, gamma-glutamyltransferase, glutamic oxaloacetic transaminase, hemoglobin, international normalized ratio, lactate dehydrogenase, magnesium, hematocrit, prothrombin time, partial thromboplastin time, mean corpuscular hemoglobin, mean corpuscular volume, mean corpuscular hemoglobin concentration
Surgery characteristics (20)	Aortic cross-clamp time, cardiopulmonary bypass time, time in operation theatre, surgery procedure (from logistic regression text model, see Supplementary Note 1)
Vital signs (8)	Systolic, mean and diastolic arterial pressure, central venous pressure, heart frequency, pulse, body temperature, oxygen saturation
Arterial blood gas values (BGA) (15)	Base excess, bicarbonate, glucose, hemoglobin, oxygen saturation, partial pressure of carbon dioxide and oxygen, total carbon dioxide, pH level, potassium, sodium, calcium, lactate, carboxyhemoglobin, oxyhemoglobin
Fluid output (2)	Bleeding Rate, urine flow rate
Nephrotoxic agents (22)	Allopurinol, Aminoglycosides, Amphotericin B, Antiplatelet agents (clopidogrel, ticlopidine), Benzodiazepines, Cephalosporins, Cyclosporine, Haloperidol, Ketamine, Nonsteroidal anti-inflammatory drugs, Paracetamol, Penicillines, Proton pump inhibitors, Pyrazolone derivatives, Quinolones, Ranitidine, Rifampin, Sulfonamides, Tacrolimus, (Val-)/Ganciclovir, Aciclovir, Vancomycin Red Blood Cell Transfusions

The last creatinine/urea value before surgery was used as a baseline. If there was none available in the five days before surgery, we used the first postoperative value.

We observed that urine output was sometimes incompletely documented on normal wards. As this could lead to false-positive AKI diagnoses we considered urine values reliable only when they were recorded in the operation theatre, the recovery room or the ICU. Thus, on normal wards AKI was only defined by the creatinine criterion whereas in the recovery room or the ICU both AKI criteria (creatinine and urine) were used.

EHR systems are often designed with billing and revision purposes in mind, making certain retrospective therapeutic analyses difficult to conduct due to missing information^48^. In our case, the type of operation that patients underwent was available partly in unstructured textual and partly in categorical form. To access both types of data, we developed a separate set of bag-of-words logistic regression models that predicted the type of operation based on unstructured text describing the operation procedures. As explanatory variables we used all single words or abbreviations that occurred in the pool of text information in its training set. The probability of a specific surgery type *Y*_*i*_ (*i* = 1, 2, …, 17) was given by

1 $$P(Y_i = 1) = \frac{{\mathrm{exp}(\beta _0 + \beta _1x_1 + \beta _2x_2 + ...)}}{{1 + \mathrm{exp}(\beta _0 + \beta _1x_1 + \beta _2x_2 + ...)}}$$

where *x*_*j*_, denotes a count variable indicating how often word/abbreviation *j* occurred in a patient’s surgery procedure description (*j* = 1, 2, …, no. distinct words/abbreviations). For further information see Supplementary Note 1, Supplementary Tables 9 and 10.

Time sequences with 15-min intervals of all features served as input to our model.

Except for the nephrotoxic agents, missing values were filled by forward imputation. If no precedent value was available, static default values defined by a clinical expert were imputed (one value per feature). The same default values were used for all patients and they were imputed programmatically. They are shown in Supplementary Table 11.

It is extremely difficult to determine the exact effect duration of a drug due to varying excipients, dosages, drug combinations, application types and patient conditions. Therefore, the administration of a drug was considered as an event. For each nephrotoxic agent class in Table 5 a binary feature was created and its value was set to 1 only at the single time slice immediately following the administration of the drug.

Except for the operation types all continuous features were then scaled as follows^49^:

2 $$X_{\mathrm{scaled}} = \frac{{X - \mu (X_{\mathrm{train}})}}{{IQR(X_{\mathrm{train}})}}$$

where *μ(X*_train_*)* denotes the median and *IQR(X*_train_*)* the IQR of the feature $X$ in the training set. In total, the model was built on a data matrix of 36,244,608 single data points.

For patient selection, preprocessing of features and imputation of missing data, we used R v3.3.3 (R Core Team (2017). R: A language and environment for statistical computing. R Foundation for Statistical Computing, Vienna, Austria. URL https://www.R-project.org/) and Python v3.6.7 (The Python Software Foundation, Beaverton, OR) with modules IPython^50^ (v7.5.0), Matplotlib^51^ (v3.1.0), Scikit-learn^52^ (v0.19.1), Pandas^53^ (v0.24.2) and Numpy^54^ (v1.16.2).

### Modeling

**Fig. 5:**
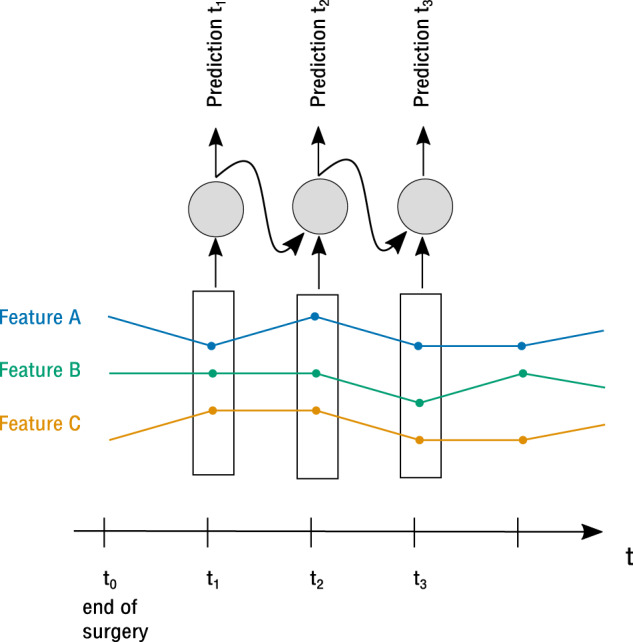
Architecture of a recurrent neural network (RNN). At each time step, the model receives the current time slice data as input as well as the own output from the preceding time step. The features are captured in a truly sequential fashion.

In contrast to classical prediction models such as logistic regression, RNNs are able to capture the temporal development of features in a truly sequential fashion as they incorporate information about preceding time steps, links between single timesteps and a direct indicator of the current position in the timeline (see Fig. 5).

We constructed a set of RNNs with different architectures (preceding convolutional layer, different cell types) which allow to process dynamic temporal information.

Hyperparameter tuning was performed on the training set using fivefold cross-validation with balanced class proportions in each fold. We used the Adam optimizer^55^ with a fixed learning rate of 0.001. The hyperparameter configurations leading to the highest overall AUC on cross-validation folds of the training set were chosen as final models.

As the parameters of an RNN depend on their initialization and the order in which the training instances are presented, 10 final models with the same hyperparameters but different initializations were trained on the training set. Our final model comprised a uniform ensemble of the 10 constituent models.

For the modeling process we used Python v3.6.7 (The Python Software Foundation, Beaverton, OR) with modules Tensorflow^56^, IPython^50^ (v7.5.0), Matplotlib^51^ (v3.1.0), Scikit-learn^52^ (v0.19.1), Pandas^53^ (v0.24.2) and Numpy^54^ (v1.16.2).

### Measuring RNN performance

We measured the performance of the RNN on an independent test set. No instance of this test set was used for training of the final model. We calculated AUC, precision-recall-AUC (PR_AUC), accuracy, sensitivity, specificity, PPV, negative predictive value (NPV), false-positive rate (FPR) and the F_1_-score to measure prediction correctness.

In addition, we calculated the mean of the Brier score^57^—or mean squared error—of each patient ($\overline {\mathrm{MSE}} _{\mathrm{pat}}$)—a measure of accuracy of predictions, without the need for a set threshold.

A single patient’s Brier score—or mean squared error—is calculated as follows:

3 $${\mathrm{MSE}}_{\mathrm{pat}} = 1/ts_j\mathop {\sum}\limits_{i = 0}^{ts_j} {(y_{ji} - y_{jt})^2}$$

where *ts*_*j*_ is the number of timesteps, *y*_*ji*_ the prediction at time step *i* and *y*_*jt*_ the true label of patient *j*.

The $\overline {\mathrm{MSE}} _{\mathrm{pat}}$ ranges from 0 to 1, with value 0 meaning perfect prediction and 1 meaning worst prediction. Random guessing (always predicting 50%) would result in a $\overline {\mathrm{MSE}} _{\mathrm{pat}}$ of 0.25. In contrast to the metrics mentioned above, the $\overline {\mathrm{MSE}} _{\mathrm{pat}}$ is independent of the individual observation length of a patient and the resulting number of predictions per patient.

We adjusted the threshold for positive class prediction until a fixed sensitivity of 0.85 on cross-validation folds in the training set was reached (threshold = 0.41).

Our model predicted the risk of developing AKI every 15 min after the initial surgery. The predictions of an individual patient can be regarded as a cluster of usually highly correlated data. We therefore had to adjust the CIs of our model’s metrics. We calculated the 95% CI of each metric *X* as follows:

$$X + - 1.96\sigma (X)$$

with a standard error $\sigma (X)$ of variable *X* of

4 $$\sigma (X) = \sqrt {\frac{{X(1 - X)}}{{n_{eff}}}}$$

To account for intracluster correlation, our sample size *n* was adjusted, resulting in an effective sample size of^58,59^

5 $$n_{\mathrm{eff}} = \frac{n}{{\mathrm{DE}}} = \frac{{\mathop {\sum }\nolimits_{i = 1}^k \mathop {\sum }\nolimits_{j = 1}^{m_i} 1}}{{\mathrm{DE}}}$$

where *k* is the number of patients and *m*_*i*_ the number of time steps of patient *i*. DE denotes the design effect, also called variance inflation factor, and can be calculated as follows^60^:

6 $${\mathrm{DE}} = \frac{{\bar mk}}{{\mathop {\sum }\nolimits_{i = 1}^k \frac{{m_i}}{{1 + (m_i - 1){\mathrm{ICC}}}}}}$$

with ICC as the intracluster correlation coefficient. The ICC was calculated using the R package ICC^61^ (v2.3.0).

### Comparing RNN vs. human performance

We set up an experiment to compare the performance of our RNN against that of experienced physicians (see Fig. 1). For each patient in the test set, a quasi-random point in time in their observation period was chosen, further denoted as the ‘prediction point’. In contrast to real uniform random samples, which tend to form clusters and contain regions without any points at all, quasi-random sequences reduce the probability of cluster formation while still being uniformly distributed^62,63^. This method prevented us from accidentally exclusively sampling prediction points from e.g. the first half of the patients’ observation periods.

At each prediction point, a physician and the RNN had to predict whether a patient would develop AKI within the first 7 days after surgery.

All time series information up to the ‘prediction point’ was graphically displayed for the physicians to mimic the electronic patient chart—although here not in 15-min intervals but in the originally recorded time resolution (up to 1 min).

To create a realistic setting, physicians not only received information about nephrotoxic agents, but of all administered drugs. In addition, the surgery type was given to them as unstructured text manually extracted from physicians’ notes. This information was not available to the RNN model. Physicians were explicitly informed about the incidence rate of 50% AKI in our test set.

A physician as well as the RNN made a probability prediction r of the development of AKI for each patient at the respective prediction point. In addition, the physicians made a binary decision (development of AKI: yes/no).

We asked 14 physicians to participate in our study, 10 of whom agreed (response rate = 0.71). All had to meet the selection criteria of ≥5 years of clinical experience and ≥1 year of work experience on a cardiothoracic ICU. From the 10 volunteers we selected seven physicians with different levels of expertise (senior resident up to senior consultant) to create a most realistic setting. Their working experience on a cardiothoracic ICU ranged from at least one year up to several years. None of the participating physicians were specialists in nephrology as nephrologists are usually not constantly available on an ICU. Each physician made predictions for 50 different patients.

### Statistical analysis

The initial aim of our study was to show that the RNN is not inferior to experienced physicians in the prediction of AKI. For both, RNN and physicians, the predictive quality of each probability prediction r was measured by a score S as follows:

$${\mathrm{S}} = {\mathrm{r}},{\mathrm{if}}\,{\mathrm{the}}\,{\mathrm{patient}}\,{\mathrm{developed}}\,{\mathrm{AKI}}$$

$${\mathrm{S}} = 1 - {\mathrm{r}},{\mathrm{if}}\,{\mathrm{the}}\,{\mathrm{patient}}\,{\mathrm{did}}\,{\mathrm{not}}\,{\mathrm{develop}}\,{\mathrm{AKI}}$$

A prior investigation of the RNN’s predictions had shown that S was non-normally distributed. Thus, for sample size calculation and power analysis we considered the transformed score *X*, which was approximately normally distributed:

7 $$X = - {\mathrm{log}}( - {\mathrm{log}}(S))$$

We assumed that *X* of the physicians’ predictions would also be normally distributed.

Based on a significance level of *α* = 0.025, a power of at least 80% and a non-inferiority margin of *δ* = 0.3 (this corresponds to a non-inferiority margin of 5.5% for sensitivity + specificity), we obtained a sample size of *N* = 350.

Both, for RNN and physicians, we calculated AUC, PR_AUC, brier score, accuracy, sensitivity, specificity, PPV, NPV, FPR and F_1_-score. We set the threshold for positive class prediction to 0.5 as this was also the threshold in the physicians’ predictions that corresponded to the ‘yes/no’-classification. We calculated CIs for all metrics as described in Section ‘Measuring RNN Performance’ whereas the effective sample size was *n*_eff_ = *n* = 350 as there was no clustering.

For the statistical comparison of S between RNN and physicians we applied a paired t-test. We used DeLong’s^64^ method to compare the two correlated ROC curves using the R package pROC^65^ (v1.9.1). In addition, we investigated the calibration of both, physicians’ and RNN’s predictions, with the Hosmer-Lemeshow-Test using the R package ResourceSelection^66^ (v0.3-2). All three comparisons mentioned above were tested on a significance level of *α* = 0.05.

### Reporting summary

Further information on research design is available in the Nature Research Reporting Summary linked to this article.

## Supplementary information

Supplementary Information

Reporting Summary

## Data Availability

The EHR data used in this study contain protected health information (PHI) and cannot be published for reasons of data protection. The dataset may be available from the German Heart Center Berlin subject to ethical approvals.

## Code availability

The source code of this publication may be obtained from the corresponding author upon request. To enable independent replication, we describe the experiments and implementation in detail in the ‘Methods’ section and in the Supplementary Information.

## References

[1] Chertow GM, Levy EM, Hammermeister KE, Grover F, Daley J (1998). Independent association between acute renal failure and mortality following cardiac surgery 12. Am. J. Med..

[2] Hobson CE (2009). Acute kidney injury is associated with increased long-term mortality after cardiothoracic surgery. Circulation.

[3] Mandelbaum T (2011). Outcome of critically ill patients with acute kidney injury using the Acute Kidney Injury Network criteria. Crit. Care Med..

[4] Ympa YP, Sakr Y, Reinhart K, Vincent J-L (2005). Has mortality from acute renal failure decreased? A systematic review of the literature. Am. J. Med..

[5] Hobson C (2015). Cost and mortality associated with postoperative acute kidney injury. Ann. Surg..

[6] Silver SA, Long J, Zheng Y, Chertow GM (2017). Cost of acute kidney injury in hospitalized patients. J. Hosp. Med..

[7] Silver SA, Chertow GM (2017). The economic consequences of acute kidney injury. Nephron.

[8] Khwaja A (2012). KDIGO clinical practice guidelines for acute kidney injury. Nephron Clin. Pract..

[9] Spanuchart I, Cheungpasitporn W, Thongprayoon C, Ratanapo S, Srivali N (2015). Off-pump versus on-pump coronary artery bypass surgery: an updated meta-analysis of randomized controlled trials on acute kidney injury and mortality outcomes. J. Am. Coll. Cardiol..

[10] Seabra VF, Alobaidi S, Balk EM, Poon AH, Jaber BL (2010). Off-pump coronary artery bypass surgery and acute kidney injury: a meta-analysis of randomized controlled trials. Clin. J. Am. Soc. Nephrol..

[11] Mao H (2014). Cardiac surgery-associated acute kidney injury. Blood Purif..

[12] Wang Y, Bellomo R (2017). Cardiac surgery-associated acute kidney injury: risk factors, pathophysiology and treatment. Nat. Rev. Nephrol..

[13] Faubel S, Shah PB (2016). Immediate consequences of acute kidney injury: the impact of traditional and nontraditional complications on mortality in acute kidney injury. Adv. Chronic Kidney Dis..

[14] Hsia CCW, Ravikumar P, Ye J (2017). Acute lung injury complicating acute kidney injury: a model of endogenous αKlotho deficiency and distant organ dysfunction. Bone.

[15] Mehta RL (2011). Sepsis as a cause and consequence of acute kidney injury: Program to Improve Care in Acute Renal Disease. Intensive Care Med..

[16] Coca SG, Singanamala S, Parikh CR (2012). Chronic kidney disease after acute kidney injury: a systematic review and meta-analysis. Kidney Int..

[17] Balasubramanian G (2011). Early nephrologist involvement in hospital-acquired acute kidney injury: a pilot study. Am. J. Kidney Dis..

[18] Costa e Silva VT (2013). Nephrology referral and outcomes in critically ill acute kidney injury patients. PLoS ONE.

[19] Meersch M (2017). Prevention of cardiac surgery-associated AKI by implementing the KDIGO guidelines in high risk patients identified by biomarkers: the PrevAKI randomized controlled trial. Intensive Care Med..

[20] Huen SC, Parikh CR (2012). Predicting acute kidney injury after cardiac surgery: a systematic review. Ann. Thorac. Surg..

[21] Chertow GM (1997). Preoperative renal risk stratification. Circulation.

[22] Thakar CV, Arrigain S, Worley S, Yared J-P, Paganini EP (2005). A clinical score to predict acute renal failure after cardiac surgery. J. Am. Soc. Nephrol..

[23] Mehta RH (2006). Bedside tool for predicting the risk of postoperative dialysis in patients undergoing cardiac surgery. Circulation.

[24] Palomba H, de Castro I, Neto ALC, Lage S, Yu L (2007). Acute kidney injury prediction following elective cardiac surgery: AKICS Score. Kidney Int..

[25] Aronson S (2007). Risk index for perioperative renal dysfunction/failure: critical dependence on pulse pressure hypertension. Circulation.

[26] Wijeysundera DN (2007). Derivation and validation of a simplified predictive index for renal replacement therapy after cardiac surgery. JAMA.

[27] Halford GS, Baker R, McCredden JE, Bain JD (2005). How many variables can humans process?. Psychol. Sci..

[28] Thottakkara P (2016). Application of machine learning techniques to high-dimensional clinical data to forecast postoperative complications. PLoS ONE.

[29] Bihorac A (2019). MySurgeryRisk: development and validation of a machine-learning risk algorithm for major complications and death after surgery. Ann. Surg..

[30] Koyner JL, Adhikari R, Edelson DP (2016). Development of a multicenter ward–based AKI prediction model. Clin. J. Am. Soc. Nephrol..

[31] Koyner JL, Carey KA, Edelson DP, Churpek MM (2018). The development of a machine learning inpatient acute kidney injury prediction model. Crit. Care Med..

[32] Cheng P, Waitman LR, Hu Y, Liu M (2017). Predicting inpatient acute kidney injury over different time horizons: how early and accurate?. AMIA Annu. Symp. Proc..

[33] Mohamadlou H (2018). Prediction of acute kidney injury with a machine learning algorithm using electronic health record data. Can. J. Kidney Health Dis..

[34] Tomašev N (2019). A clinically applicable approach to continuous prediction of future acute kidney injury. Nature.

[35] Meyer A (2018). Machine learning for real-time prediction of complications in critical care: a retrospective study. Lancet Respir. Med..

[36] Hosmer, D. W., Jr., Lemeshow, S. & Sturdivant, R. X. *Applied Logistic Regression* (John Wiley & Sons, 2013).

[37] Praught ML, Shlipak MG (2005). Are small changes in serum creatinine an important risk factor?. Curr. Opin. Nephrol. Hypertens..

[38] Brown JR (2007). Multivariable prediction of renal insufficiency developing after cardiac surgery. Circulation.

[39] Koyner JL, Adhikari R, Edelson DP, Churpek MM (2016). Development of a multicenter ward-based AKI prediction model. Clin. J. Am. Soc. Nephrol..

[40] Mohamadlou H (2018). Prediction of acute kidney injury with a machine learning algorithm using electronic health record data. Can. J. Kidney Health Dis..

[41] Collins GS, Reitsma JB, Altman DG, Moons KGM (2015). Transparent Reporting of a multivariable prediction model for Individual Prognosis or Diagnosis (TRIPOD): the TRIPOD statement. Ann. Intern. Med..

[42] Naughton CA (2008). Drug-induced nephrotoxicity. Am. Fam. Physician.

[43] Mazer M, Perrone J (2008). Acetaminophen-induced nephrotoxicity: pathophysiology, clinical manifestations, and management. J. Med. Toxicol..

[44] Kitano A, Motohashi H, Takayama A, Inui K-I, Yano Y (2014). Valacyclovir-Induced Acute Kidney Injury in Japanese Patients Based on the PMDA Adverse Drug Reactions Reporting Database. Drug Inf. J..

[45] Redondo-Pachon MD (2014). Acute renal failure and severe thrombocytopenia associated with metamizole. Saudi J. Kidney Dis. Transpl..

[46] Koch CG (2008). Duration of red-cell storage and complications after cardiac surgery. N. Engl. J. Med..

[47] Nuis R-J (2012). Blood transfusion and the risk of acute kidney injury after transcatheter aortic valve implantation. Circ. Cardiovasc. Interv..

[48] Johnson AEW (2016). Machine Learning and Decision Support in Critical Care. Proc. IEEE Inst. Electr. Electron. Eng..

[49] LeCun, Y. A., Bottou, L., Orr, G. B. & Müller, K.-R. in *Neural Networks: Tricks of the Trade: Second Edition* (eds. Montavon, G., Orr, G. B. & Müller, K.-R.) 9–48 (Springer Berlin Heidelberg, 2012).

[50] Perez F, Granger BE (2007). IPython: a system for interactive scientific computing. Comput. Sci. Eng..

[51] Hunter JD (2007). Matplotlib: a 2D graphics environment. Comput. Sci. Eng..

[52] Pedregosa F (2011). Scikit-learn: machine learning in Python. J. Mach. Learn. Res..

[53] McKinney W, Others. (2010). Data structures for statistical computing in python. Proc. 9th Python Sci. Conf..

[54] van der Walt S, Colbert SC, Varoquaux G (2011). The NumPy Array: A Structure for Efficient Numerical Computation. Comput. Sci. Eng..

[55] Kingma, D. P. & Ba, J. Adam: a method for stochastic optimization. Preprint at http://arxiv.org/abs/1412.6980 (2014)

[56] Abadi, M. et al. TensorFlow: large-scale machine learning on heterogeneous distributed systems, 2015. Software available from https://www.tensorflow.org/about/bib.

[57] BRIER & W, G. Verification of Forecasts Expressed in terms of probability. *Monthey Weather Rev.* **78**, 1–3 (1950).

[58] Kalton, G., Michael Brick, J. & Lê, T. Chapter VI Estimating components of design effects for use in sample design. http://citeseerx.ist.psu.edu/viewdoc/summary?doi:10.1.1.522.3221.

[59] Gonzalez, E. J. & Foy, P. *Third International Mathematics and Science Study, Technical Report: Estimation of sampling variability, design effects, and effective sample sizes*. p. 87 (II, Boston College Chestnut Hill, Massachusetts, USA, 1997).

[60] Kerry SM, Bland JM (2001). Unequal cluster sizes for trials in English and Welsh general practice: implications for sample size calculations. Stat. Med..

[61] Wolak ME, Fairbairn DJ, Paulsen YR (2012). Guidelines for estimating repeatability. Methods Ecol. Evol..

[62] Press, W. H., Teukolsky, S. A., Vetterling, W. T. & Flannery, B. P. In *Numerical Recipes in FORTRAN: The Art of Scientific Computing* **2**, 299–306 (Cambridge University Press, 1992).

[63] Weyl H (1916). Über die Gleichverteilung von Zahlen mod. Eins. Math. Ann..

[64] DeLong ER, DeLong DM, Clarke-Pearson DL (1988). Comparing the areas under two or more correlated receiver operating characteristic curves: a nonparametric approach. Biometrics.

[65] Robin X (2011). pROC: an open-source package for R and S+ to analyze and compare ROC curves. BMC Bioinforma..

[66] Lele, S. R., Keim, J. L. & Solymos, P. ResourceSelection: resource selection (probability) functions for use-availability data. *R package version* 3–2 (2017). Software available at https://cran.r-project.org/src/contrib/Archive/ResourceSelection/.

[67] Jiang W (2016). Dynamic predictive scores for cardiac surgery–associated acute kidney injury. J. Am. Heart Assoc..

