# Supplementary material for: Deep-learning-based real-time prediction of acute kidney injury outperforms human predictive performance
Source: NPJ Digit Med. 2020 Oct 26;3:139. doi: 10.1038/s41746-020-00346-8 (PMC7588492; doi:10.1038/s41746-020-00346-8)
Supplement: Supplementary file 1 — Supplementary Information [file 41746_2020_346_MOESM1_ESM.pdf]

Supplementary Information

The PDF file includes:

Supplementary Results 1: Performance metrics for imbalanced test set
Supplementary Note 1: Text model for surgery type
Supplementary Figure 1: Patient with highly fluctuating prediction values and correlating features
Supplementary Figure 2: Patient without acute kidney injury but high prediction values (false-positive)
Supplementary Figure 3: Patient with acute kidney injury but low prediction values (false-negative)
Supplementary Table 1: Baseline comparison between AKI- and non-AKI cases in the training set
Supplementary Table 2: Baseline comparison between AKI- and non-AKI cases in the balanced test set
Supplementary Table 3: Baseline comparison between AKI- and non-AKI cases in the imbalanced test set
Supplementary Table 4: Baseline comparison between AKI- and non-AKI cases in the whole study population before matching AKI- and non-AKI cases
Supplementary Table 5: Model performance metrics for balanced test set (n = 350 admissions/patients) of a model with only creatinine as feature
Supplementary Table 6: Model performance metrics for balanced test set (n = 350 admissions/patients) of a model without creatinine and glomerular filtration rate as features
Supplementary Table 7: Model performance metrics of an imbalanced test set
Supplementary Table 8: Baseline characteristics across the training and the test set
Supplementary Table 9: Performance metrics of the text model for prediction of the type of surgery
Supplementary Table 10: Predicted frequencies of the types of surgery in the training and test set
Supplementary Table 11: Default values used for imputation

Supplementary Results 1: Performance metrics for imbalanced test set

We additionally tested our recurrent neural network (RNN) on a test set with the real incidence rate (10%) of our original study population and a sample size of 1,945 admissions. The results are shown in Supplementary Table 7. (As the calculation of the intracluster coefficient (ICC) exceeded the computational capacity of our servers, we used the ICC of the balanced test set for the calculation of the confidence intervals.)

Overall, the AUC was approximately 5 % lower than in the balanced test set. As the RNN was trained on a balanced test set (incidence rate 50%), not surprisingly, the false-positive rate increased when the model was tested on a set with lower incidence rate. Accordingly, the negative predictive value reached > 0.99.

Supplementary Note 1: Text model for surgery type

The information about the type of original operation we used in our study, was available at the beginning of patients' observation time partly in unstructured textual and partly in categorical form. When entering the surgery procedures into the database, physicians could either choose to type in free text or to select predefined procedures.

To integrate both types of data into our future models, we developed a separate set of logistic regression models that predicted the type of operation based on the available text information. As features we used all single words or abbreviations that occurred in the pool of text information. We defined 17 surgery type classes as labels that were extracted from the codes of the official classification of operational procedures in Germany (OPS). These codes were often generated for billing purposes after a patient was discharged. Combinations of single procedure types were possible.

We trained the model on the text information of 12,787 admissions and evaluated it on 986 independent test examples. As performance metrics we calculated sensitivity, specificity, positive predictive value and negative predictive value for a threshold of 0.5.

Supplementary Table 9 shows all included surgery type classes and the respective performance metrics across the test set of the text model. The model performed very well for clearly defined operation types. For most of them there was also a categorical option the physician could select when entering the surgery type. Classes comprising different types of surgery (e.g. ‘Other major operation’) or having very few training examples (e.g. ‘Transapical TMVI’) were more often classified incorrectly.

The 17 operation classes served as continuous rather than categorical features in our AKI prediction model. Their values corresponded to the predicted probabilities from the respective logistic regression text model. With the future goal of integrating our models in a real clinical setting we only used text information that was already available upon admission to the ICU or recovery room. The surgery type of patients for whom the text information was entered later into the database was considered as undetermined and all their operation class features were set to 0.

Supplementary Table 10 shows the number of patients with available text data upon admission to the ICU or recovery room and probability values >= 50% for each of the included operation classes respectively across the training and the test set of our RNN.

Supplementary Figures

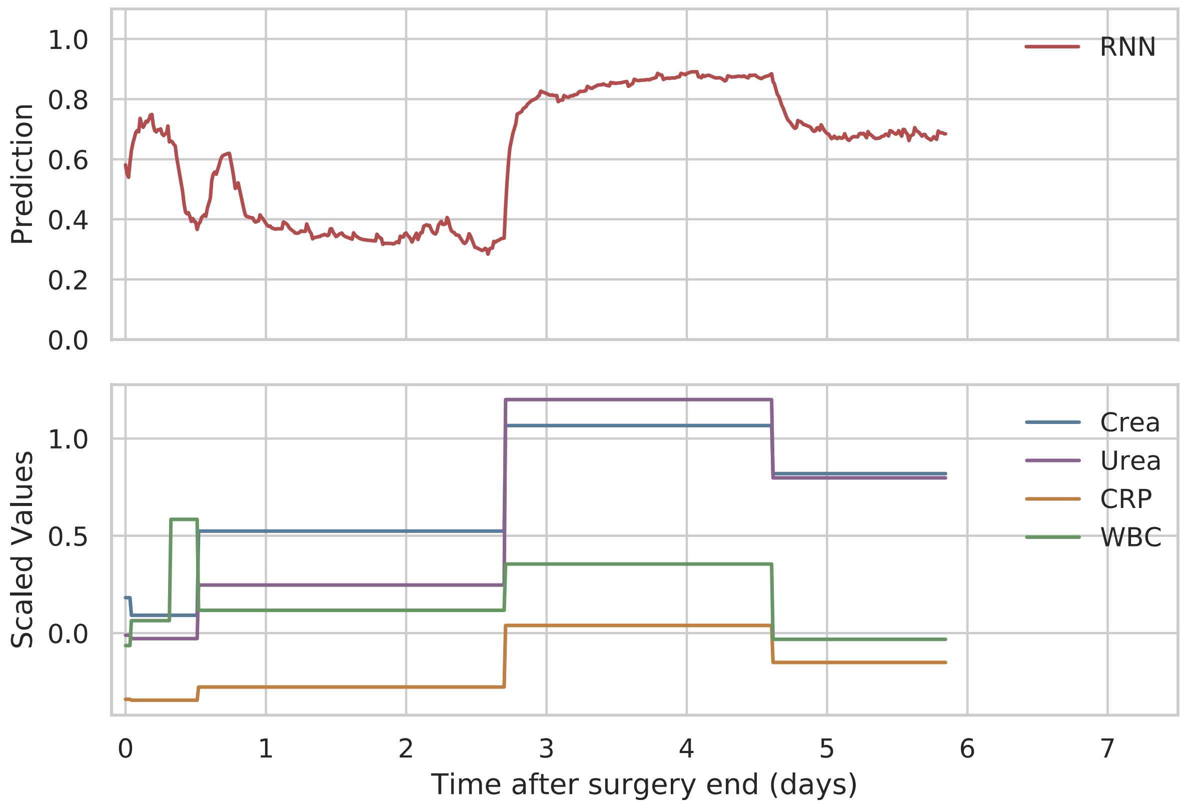

Supplementary Figure 1. Patient with highly fluctuating prediction values (top) and correlating features (bottom). For better visualization, not the absolute, but the scaled variables are displayed (see equation (2) in the main article). A value of 0 is equal to the median of the respective variable in the training set. Between day 2 and 3 an abrupt increase of the prediction can be noted. It seems to reflect the sudden increase of creatinine, urea, C-reactive protein (CRP) and white blood count (WBC). RNN = recurrent neural network.

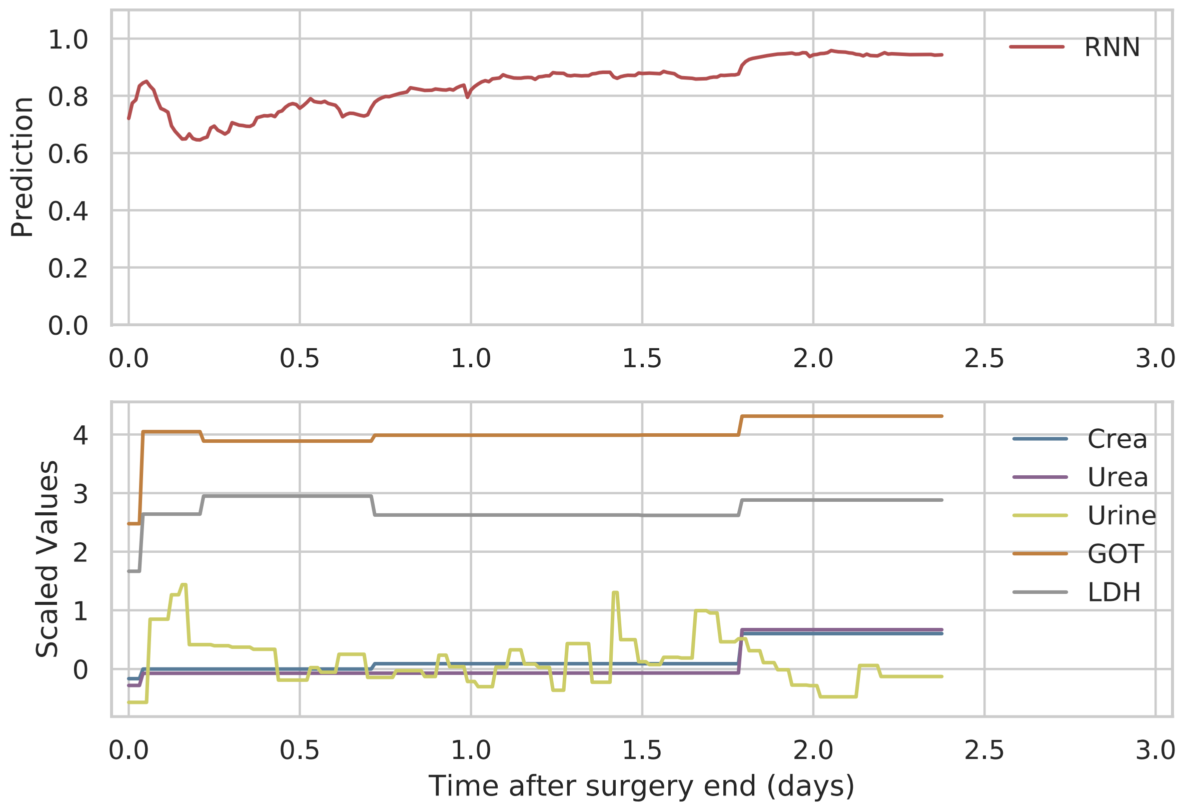

Supplementary Figure 2. Patient without acute kidney injury but high prediction values (false-positive) (top). For better visualization, not the absolute, but the scaled variables are displayed (see equation (2) in the main article). A value of 0 is equal to the median of the respective variable in the training set. Glutamic oxaloacetic transaminase (GOT) and lactate dehydrogenase (LDH) showed high levels, but creatinine, urea and urine flow were normal (bottom).

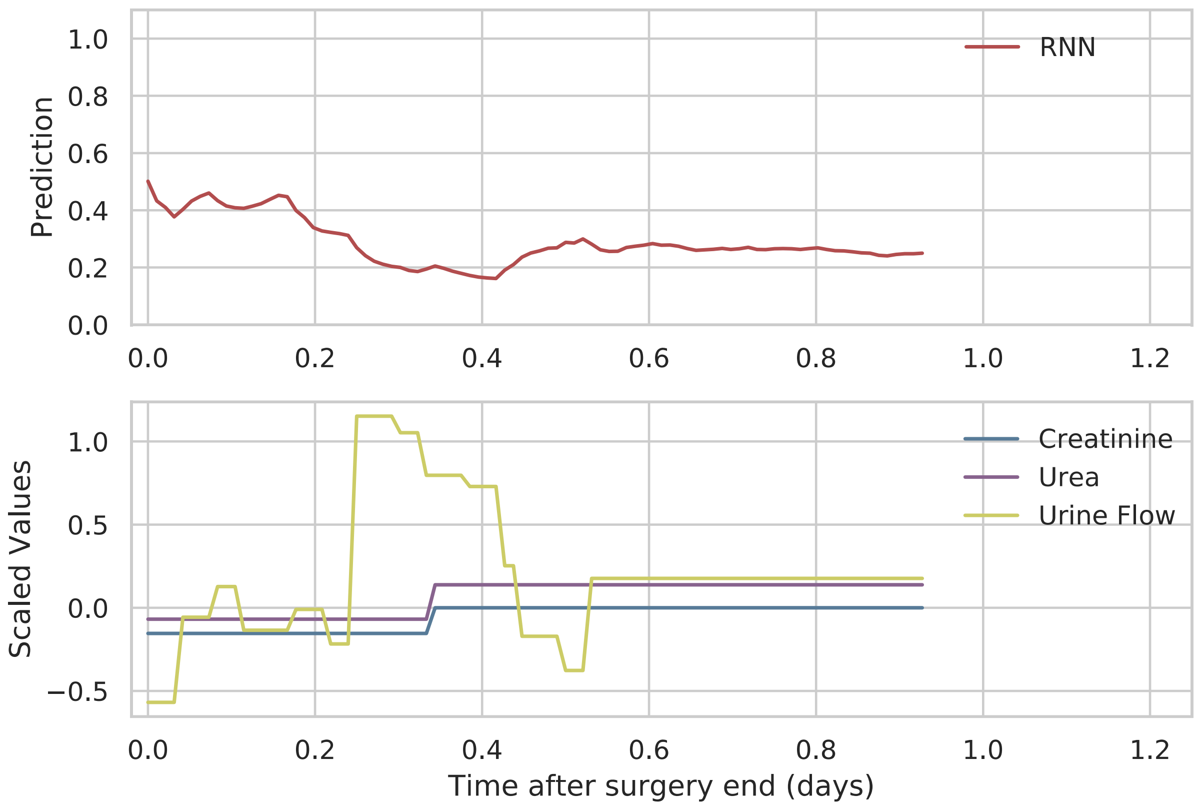

Supplementary Figure 3. Patient with acute kidney injury (AKI) but low prediction values (false-negative) (top). For better visualization, not the absolute, but the scaled variables are displayed (see equation (2) in the main article). A value of 0 is equal to the median of the respective variable in the training set. Creatinine, urea and urine flow were normal (bottom). At the end of the prediction window there was a sudden increase of creatinine (not shown here as the observation period ended with the fulfilled definition of AKI). There were no clear hints in the data that pointed out the development of AKI.

Supplementary Tables

Supplementary Table 1. Baseline comparison between AKI- and non-AKI cases in the training set. AKI = acute kidney injury, OR = odds ratio, CPB = cardio-pulmonary bypass.

	All patients	non-AKI	AKI	Odds Ratio	p-value (OR)	p-value (overall)
	N=2224	N=1112	N=1112			
Male, no. (%)	1424 (64.0%)	643 (57.8%)	781 (70.2%)	0.58 [0.49;0.69]	<0.001	<0.001
Age [years] mean ± std	68.5±14.2	72.9±12.8	64.2±14.3	0.95 [0.95;0.96]	<0.001	<0.001
Baseline creatinine [mg/dl] mean ± std	1.25±0.64	1.08±0.45	1.41±0.75	2.64 [2.23;3.12]	<0.001	<0.001
Baseline urea [mg/dl] mean ± std	52.1±31.1	46.5±26.3	57.7±34.4	1.01 [1.01;1.02]	<0.001	<0.001
Time in operation theatre [min] mean ± std	350±210	274±154	426±231	1.00 [1.00;1.01]	<0.001	<0.001
On-pump procedures, no. (%)	1134 (51.0%)	340 (30.6%)	794 (71.4%)	5.66 [4.72;6.80]	<0.001	<0.001
CBP time [min] mean ± std	139±90.1	119±70.6	148±96.0	1.00 [1.00;1.01]	<0.001	<0.001
Aortic cross clamp time [min] mean ± std	81.4±58.6	70.5±33.3	86.5±66.6	1.01 [1.00;1.01]	<0.001	<0.001

Supplementary Table 2. Baseline comparison between AKI- and non-AKI cases in the balanced test set. AKI = acute kidney injury, OR = odds ratio, CPB = cardio-pulmonary bypass.

	**All patients**	**non-AKI**	**AKI**	**Odds Ratio**	**p-value (OR)**	**p-value (overall)**
	N=350	N=175	N=175			
Male, no. (%)	233 (66.6%)	113 (64.6%)	120 (68.6%)	0.84 [0.53;1.31]	0.431	0.497
Age [years] mean ± std	68.7±13.9	72.0±13.5	65.5±13.6	0.96 [0.95;0.98]	<0.001	<0.001
Baseline creatinine [mg/dl] mean ± std	1.21±0.63	1.06±0.47	1.36±0.72	2.42 [1.61;3.64]	<0.001	<0.001
Baseline urea [mg/dl] mean ± std	49.8±31.4	44.9±25.4	54.7±35.9	1.01 [1.00;1.02]	0.005	0.004
Time in operation theatre [min] mean ± std	347±190	279±148	415±202	1.01 [1.00;1.01]	<0.001	<0.001
On-pump procedures, no. (%)	177 (50.6%)	56 (32.0%)	121 (69.1%)	4.73 [3.03;7.49]	<0.001	<0.001
CBP time [min] mean ± std	146±94.7	112±53.5	162±105	1.01 [1.00;1.01]	0.001	<0.001
Aortic cross clamp time [min] mean ± std	82.0±54.5	71.4±38.5	87.0±60.1	1.01 [1.00;1.02]	0.104	0.060

Supplementary Table 3. Baseline comparison between AKI- and non-AKI cases in the imbalanced test set. AKI = acute kidney injury, OR = odds ratio, CPB = cardio-pulmonary bypass.

	All patients	non-AKI	AKI	Odds Ratio	p-value (OR)	p-value (overall)
	N=1945	N=1749	N=196			
Male, no. (%)	1326 (68.2%)	1192 (68.2%)	134 (68.4%)	0.99 [0.72;1.36]	0.958	1.000
Age [years] mean ± std	66.7±12.8	66.9±12.7	65.3±13.6	0.99 [0.98;1.00]	0.110	0.130
Baseline creatinine [mg/dl] mean ± std	1.10±0.48	1.06±0.42	1.41±0.79	2.77 [2.19;3.49]	<0.001	<0.001
Baseline urea [mg/dl] mean ± std	44.6±25.5	43.3±23.5	56.4±36.6	1.01 [1.01;1.02]	<0.001	<0.001
Time in operation theatre [min] mean ± std	324±161	316±152	400±212	1.00 [1.00;1.00]	<0.001	<0.001
On-pump procedures, no. (%)	1161 (59.7%)	1032 (59.0%)	129 (65.8%)	1.34 [0.98;1.83]	0.064	0.077
CBP time [min] mean ± std	120±73.4	115±66.9	162±104	1.01 [1.00;1.01]	<0.001	<0.001
Aortic cross clamp time [min] mean ± std	72.3±37.7	70.8±34.2	85.8±59.5	1.01 [1.00;1.01]	<0.001	0.012

Supplementary Table 4. Baseline comparison between AKI- and non-AKI cases in the whole study population before matching AKI- and non-AKI cases. AKI = acute kidney injury, OR = odds ratio, CPB = cardio-pulmonary bypass.

	**All patients**	**non-AKI**	**AKI**	**Odds Ratio**	**p-value (OR)**	**p-value (overall)**
	N=12978	N=11670	N=1308			
Male, no. (%)	8663 (66.8%)	7748 (66.4%)	915 (70.0%)	0.85 [0.75;0.96]	0.009	0.010
Age [years] mean ± std	66.7±13.2	67.0±13.1	64.3±14.2	0.99 [0.98;0.99]	<0.001	<0.001
Baseline creatinine [mg/dl] mean ± std	1.10±0.47	1.06±0.41	1.41±0.76	2.99 [2.72;3.28]	<0.001	<0.001
Baseline urea [mg/dl] mean ± std	44.9±25.2	43.5±23.5	57.5±34.7	1.02 [1.01;1.02]	<0.001	<0.001
Time in operation theatre [min] mean ± std	326±178	315±168	422±228	1.00 [1.00;1.00]	<0.001	<0.001
On-pump procedures, no. (%)	7981 (61.5%)	7058 (60.5%)	923 (70.6%)	1.57 [1.38;1.78]	<0.001	<0.001
CBP time [min] mean ± std	117±65.0	113±58.3	150±97.2	1.01 [1.01;1.01]	<0.001	<0.001
Aortic cross clamp time [min] mean ± std	71.9±37.6	70.2±32.3	86.4±65.6	1.01 [1.01;1.01]	<0.001	<0.001

Supplementary Table 5. Model performance metrics for balanced test set (n = 350 admissions/patients) of a model with only creatinine as feature. AUC = area under curve, PR_AUC = precision-recall AUC, $\overline{MSE}_{pat}$ = mean of the brier score of each patient, Acc = accuracy, Sens = sensitivity, Spec = specificity, F1 = F_1-score, FPR = false-positive rate, NPV = negative predictive value, PPV = positive predictive value, CI = confidence interval. The threshold for positive/negative class prediction was set to 0.41.

Threshold-independent metrics, (95 % CI)			Threshold dependent metrics, (95 % CI)						
**AUC**	**PR_AUC**	$\overline{MSE}_{pat}$	**Acc**	**Sens**	**Spec**	**F1**	**FPR**	**NPV**	**PPV**
0.805 (0.768 - 0.842)	0.797 (0.759 - 0.835)	0.176 (0.136 - 0.216)	0.731 (0.689 - 0.772)	0.756 (0.698 - 0.814)	0.707 (0.647 - 0.766)	0.733 (0.681 - 0.785)	0.293 (0.234 - 0.353)	0.752 0.694 - 0.811)	0.711 (0.651 - 0.770)

Supplementary Table 6. Model performance metrics for balanced test set (n = 350 admissions/patients) of a model without creatinine and glomerular filtration rate as features. AUC = area under curve, PR_AUC = precision-recall AUC, $\overline{MSE}_{pat}$ = mean of the brier score of each patient, Acc = accuracy, Sens = sensitivity, Spec = specificity, F_1-score, FPR = false-positive rate, NPV = negative predictive value, PPV = positive predictive value, CI = confidence interval. The threshold for positive/negative class prediction was set to 0.41.

Threshold-independent metrics, (95 % CI)			Threshold dependent metrics, (95 % CI)						
**AUC**	**PR_AUC**	$\overline{MSE}_{pat}$	**Acc**	**Sens**	**Spec**	**F1**	**FPR**	**NPV**	**PPV**
0.887 (0.855 - 0.919)	0.898 (0.867 - 0.928)	0.130 (0.094 - 0.165)	0.809 (0.769 - 0.849)	0.850 (0.798 - 0.901)	0.770 (0.711 - 0.830)	0.813 (0.762 - 0.863)	0.230 (0.170 - 0.289)	0.843 0.789- 0.897)	0.779 (0.722 - 0.836)

Supplementary Table 7. Model performance metrics of an imbalanced test set. The incidence rate of 10% acute kidney injury in this test set with n = 1945 admissions corresponds to that of the original study population. AUC = area under curve, PR_AUC = precision-recall AUC, $\overline{MSE}_{pat}$= mean of the brier score of each patient, Acc = accuracy, Sens = sensitivity, Spec = specificity, F1 = F_1-score, FPR = false-positive rate, NPV = negative predictive value, PPV = positive predictive value, CI = confidence interval. The threshold for positive/negative class prediction was set to 0.41, leading to a sensitivity of 0.850 on cross-validation in the training set.

**Threshold-independent metrics, mean (95 % CI)**			**Threshold dependent metrics, (95 % CI)**						
**AUC**	**PR_AUC**	$\overline{MSE}_{pat}$	**Acc**	**Sens**	**Spec**	**F1**	**FPR**	**NPV**	**PPV**
0.846 (0.831 - 0.862)	0.152 (0.137 - 0.168)	0.153 (0.137 - 0.169)	0.714 (0.695 - 0.734)	0.868 (0.790 - 0.945)	0.709 (0.689 - 0.728)	0.176 (0.147 - 0.205)	0.291 (0.271 - 0.311)	0.993 (0.989 - 0.997)	0.098 (0.075 - 0.120)

Supplementary Table 8. Baseline characteristics across the training and the test set. The baseline characteristics were well balanced across the training and the test set. AKI = acute kidney injury, CPB = cardiopulmonary bypass, IQR = interquartile range.

Variable	Training Set	Test Set
No. of admissions	2,224	350
No. of individual patients (%)	2,180 (98)	350 (100)
No. of cases with AKI (%)	1,112 (50)	175 (50)
No. of cases with AKI defined by serum creatinine criteria or initiation of dialysis (% of total AKI cases)	778 (70)	120 (69)
No. of cases with AKI defined by urine criteria (% of total AKI cases)	334 (30)	55 (31)
Length of observation period in days, median (IQR)	1.33 (0.58 - 2.36)	1.30 (0.55 - 2.13)
Age, median (IQR)	72 (60 - 79)	71 (61 -79)
Male, No. (%)	1,424 (64)	233 (67)
Baseline creatinine [mg/dl], median (IQR)	1.1 (0.83 - 1.4)	1.0 (1.0 - 1.0)
Baseline urea [mg/dl], median (IQR)	43 (32 - 62)	41 (31 - 56)
Time in operation theatre [minutes], median (IQR)	308 (208 - 450)	314 (214 - 428)
On-pump procedures, No. (%)	1134 (51)	177 (51)
Aortic cross clamp time [minutes], median (IQR)	81 (53 - 105)	77 (52 - 100)
CPB time [minutes], median (IQR)	118 (78 - 183)	122 (83 - 180)

Supplementary Table 9. Performance metrics of the text model for prediction of the type of surgery. The set of logistic regression-based text models for prediction of the type of surgery was evaluated on an individual test set (n = 986). Sensitivity, specificity, positive predictive value (PPV) and negative predictive value (NPV) are based on a threshold of 0.5.

**Surgery Type**	**Sensitivity**	**Specificity**	**PPV**	**NPV**
Valve surgery	0.921	0.985	0.932	0.982
Transcatheter aortic valve implantation (TAVI)	0.968	0.999	0.999	0.968
Endovascular TAVI	1.000	1.000	1.000	1.000
Transapical TAVI	0.867	1.000	0.998	1.000
Coronary artery bypass grafting (CABG)	0.843	0.933	0.926	0.856
Off-pump CABG	0.664	0.98	0.951	0.833
Aortic surgery	0.703	0.99	0.951	0.927
Assist device	0.875	0.999	0.994	0.977
Ventricular assist device	0.966	1.000	0.999	1.000
Extracorporeal membrane oxygenation system	0.500	1.000	0.995	1.000
Endovascular aortic stent implantation	0.889	0.999	0.995	0.976
Transplantation	1.000	1.000	1.000	1.000
Other major cardiac surgery	0.233	0.989	0.942	0.63
Isolated other major cardiac surgery	0.321	0.996	0.980	0.692
Transcatheter mitral valve implantation (TMVI)	0.714	1.000	0.998	1.000
Endovascular TMVI*	-	-	-	-
Transapical TMVI	0.286	1.000	0.995	1.000
*As there was no instance of endovascular TMVI in our test set, metrics were not calculated.				

Supplementary Table 10. Predicted frequencies of the types of surgery in the training and test set. The type of surgery used in the recurrent neural network was predicted from structured and unstructured text with a set of logistic regression-based text models. Only text information that was already available upon admission to the ICU or recovery room was used. The values indicate the number of patients with probability values >= 50 % for each of the included operation classes respectively.

**Variable**	**Training Set**	**Test Set**
Total no. of patients	2,224	350
No. of patients with available text information at the end of surgery (%)	1,102 (50)	171 (49)
Valve surgery	292	49
Transcatheter aortic valve implantation (TAVI)	270	35
Endovascular TAVI	247	33
Transapical TAVI	23	2
Coronary artery bypass grafting (CABG)	252	47
Off-pump CABG	11	0
Aortic surgery	104	16
Assist device	52	3
Ventricular assist device	47	2
Extracorporeal membrane oxygenation system	4	0
Endovascular aortic stent implantation	31	3
Transplantation	57	8
Other major cardiac surgery	21	4
Isolated other major cardiac surgery	13	3
Transcatheter mitral valve implantation (TMVI)	0	0
Endovascular TMVI	0	0
Transapical TMVI	0	0

Supplementary Table 11. Default values used for imputation.

Height	1.70 m	Cardiopulmonary bypass time	0 min
Weight	75 kg	Surgery procedure	0 for all surgery types
Phosphate	1.1 mmol/l	Systolic arterial pressure	115 mmHg
Total bilirubin	0.8 mg/dl	Mean arterial pressure	75 mmHg
Creatinine	1.0 mg/dl	Diastolic arterial pressure	60 mmHg
Urea	38 mg/dl	Central venous pressure	9 mmHg
Glomerular filtration rate	65 ml/min	Heart frequency	80 /min
Creatine kinase (CK)	212 U/l	Pulse	80 /min
CK-MB	24 U/l	Body temperature	37 °C
Red blood count,	$3.5 * 10^{9}$/µl	Oxygen saturation	98 %
White blood count	$10 * 10^{3}$/µl	Base excess	0 mmol/l
Platelets	$160 * 10^{3}$/µl	Bicarbonate	24 mmol/l
C-reactive protein	5 mg/l	Glucose	130 mg/dl
Gamma-glutamyltransferase	27 U/l	Oxygen saturation (BGA)	98 %
Glutamic oxaloacetic transaminase	49 U/l	Partial pressure of carbon dioxide	39 mmHg
Hemoglobin	10 g/dl	Partial pressure of oxygen	110 mmHg
International normalized ratio	1.3	Total carbon dioxide	57 mmol/l
Lactate dehydrogenase	300 U/l	PH level	7.37
Magnesium	0.86 mmol/l	Potassium	4.1 mmol/l
Hematocrit	32 %	Sodium	140 mmol/l
Prothrombin time	69 s	Calcium	1.2 mmol/l
Partial thromboplastin time	43 s	Lactate	11 mmol/l
Mean corpuscular hemoglobin	30 pg	Carboxyhemoglobin	1.1 %
Mean corpuscular volume	90 fl	Oxyhemoglobin	98 %
Mean corpuscular hemoglobin concentration	33 g/dl	Bleeding Rate	0 ml/h
Aortic cross-clamp time	0 min	Urine flow rate	0 ml/h
