# Supplementary material for: Deep-learning-based real-time prediction of acute kidney injury outperforms human predictive performance
Source: NPJ Digit Med. 2020 Oct 26;3:139. doi: 10.1038/s41746-020-00346-8 (PMC7588492; doi:10.1038/s41746-020-00346-8)
Supplement: Supplementary file 2 — Reporting Summary [file 41746_2020_346_MOESM2_ESM.pdf]

nature research

Corresponding author(s): Alexander Meyer

Last updated by author(s): Aug 29, 2020

Reporting Summary

Nature Research wishes to improve the reproducibility of the work that we publish. This form provides structure for consistency and transparency in reporting. For further information on Nature Research policies, see Authors & Referees and the Editorial Policy Checklist.

Statistics

For all statistical analyses, confirm that the following items are present in the figure legend, table legend, main text, or Methods section.

n/a	Confirmed	
☐	☒	The exact sample size (*n*) for each experimental group/condition, given as a discrete number and unit of measurement
☐	☒	A statement on whether measurements were taken from distinct samples or whether the same sample was measured repeatedly
☐	☒	The statistical test(s) used AND whether they are one- or two-sided<br>*Only common tests should be described solely by name; describe more complex techniques in the Methods section.*
☐	☒	A description of all covariates tested
☐	☒	A description of any assumptions or corrections, such as tests of normality and adjustment for multiple comparisons
☐	☒	A full description of the statistical parameters including central tendency (e.g. means) or other basic estimates (e.g. regression coefficient) AND variation (e.g. standard deviation) or associated estimates of uncertainty (e.g. confidence intervals)
☐	☒	For null hypothesis testing, the test statistic (e.g. *F*, *t*, *r*) with confidence intervals, effect sizes, degrees of freedom and *P* value noted<br>*Give P values as exact values whenever suitable.*
☒	☐	For Bayesian analysis, information on the choice of priors and Markov chain Monte Carlo settings
☒	☐	For hierarchical and complex designs, identification of the appropriate level for tests and full reporting of outcomes
☒	☐	Estimates of effect sizes (e.g. Cohen's *d*, Pearson's *r*), indicating how they were calculated

Our web collection on statistics for biologists contains articles on many of the points above.

Software and code

Policy information about availability of computer code

Data collection	Data collection was performed using R v3.3.3 (R Core Team (2017). R: A language and environment for statistical computing. R Foundation for Statistical Computing, Vienna, Austria. URL https://www.R-project.org/) and Python v3.6.7 (The Python Software Foundation, Beaverton, OR) with modules IPython (v7.5.0), Matplotlib (v3.1.0), Scikit-learn (v0.19.1), Pandas (v0.24.2) and Numpy (v1.16.2).
Data analysis	Data analysis was performed using R v3.3.3 (R Core Team (2017). R: A language and environment for statistical computing. R Foundation for Statistical Computing, Vienna, Austria. URL https://www.R-project.org/) and Python v3.6.7 (The Python Software Foundation, Beaverton, OR) with packages Tensorflow, IPython (v7.5.0), Matplotlib (v3.1.0), Scikit-learn (v0.19.1), Pandas (v0.24.2) and Numpy (v1.16.2).

For manuscripts utilizing custom algorithms or software that are central to the research but not yet described in published literature, software must be made available to editors/reviewers. We strongly encourage code deposition in a community repository (e.g. GitHub). See the Nature Research guidelines for submitting code & software for further information.

Data

Policy information about availability of data

All manuscripts must include a data availability statement. This statement should provide the following information, where applicable:

- Accession codes, unique identifiers, or web links for publicly available datasets
- A list of figures that have associated raw data
- A description of any restrictions on data availability

The data used in this study was collected from the electronic health record system of Deutsches Herzzentrum Berlin, Germany, between October 2012 and February 2018. It contains protected health information (PHI) and cannot be published for reasons of data protection. The dataset may be available from the German Heart Center Berlin subject to ethical approvals.

Field-specific reporting

Please select the one below that is the best fit for your research. If you are not sure, read the appropriate sections before making your selection.

☒ Life sciences ☐ Behavioural & social sciences ☐ Ecological, evolutionary & environmental sciences

For a reference copy of the document with all sections, see nature.com/documents/nr-reporting-summary-flat.pdf

Life sciences study design

All studies must disclose on these points even when the disclosure is negative.

Sample size

We included adult patients (18+) that were admitted at least once to the operating theatre for cardiothoracic surgery (15,564 admissions/13,895 patients). Please see 'Methods' for further description of the patient selection process.

The sample size calculation for our head-to-head comparison recurrent neural network vs. physicians was based on a non-inferiority design. Based on a significance level of α=0.025, a power of at least 80% and a non-inferiority margin of δ = 0.3 (this corresponds to a non-inferiority margin of 5.5% for sensitivity+specificity), we obtained a sample size of N = 350.

Data exclusions

We excluded patients without any creatinine or urine flow values, patients receiving hemodialysis before the end of the operation or having a baseline creatinine level >= 4.0mg/dl (2,322 admissions/1,487 patients).

For the 392 patients of the set eligible for validation we manually checked physicians' notes in the EHR data and consequently excluded 28 patients. Exclusion criteria were primarily insufficient documentation of the type of surgery, false recording of surgery times or notion of end-stage kidney disease in the patients' history that was not detected by automated filtering.

Replication

The 350 patients in the independent test set were randomly selected and were not correlated. Applying the model on their electronic health record data can be regarded as 350 replicas of the model.

Randomization

Each acute kidney injury (AKI)-case was assigned a control out of the non-AKI pool (11,670 admissions/11,046 patients). The controls were matched to the cases on observation length. Thus, we generated a balanced data set that we then randomly split into a training set (85%, 2,224 admissions/2,180 patients) and the remaining set (15%, 392 admissions/patients) while keeping the cases with their respective controls. After manual checking of physicians' notes in the EHR data and exclusion of 28 patients, the final 350 test patients were randomly selected. For each patient in the test set, a point in time in their observation period was chosen, where model an physicians had to make a prediction. This prediction point was chosen quasi-randomly to reduce the probability of cluster formation while keeping a uniform distribution.

Blinding

Participants in the head-to-head comparison recurrent neural network vs. physicians were blinded to the clinical cases. The incidence rate of acute kidney injury in the test set was reported to them.

Reporting for specific materials, systems and methods

We require information from authors about some types of materials, experimental systems and methods used in many studies. Here, indicate whether each material, system or method listed is relevant to your study. If you are not sure if a list item applies to your research, read the appropriate section before selecting a response.

Materials & experimental systems

n/a	Involved in the study	
☒	☐	Antibodies
☒	☐	Eukaryotic cell lines
☒	☐	Palaeontology
☒	☐	Animals and other organisms
☐	☒	Human research participants
☒	☐	Clinical data

Methods

n/a	Involved in the study	
☒	☐	ChIP-seq
☒	☐	Flow cytometry
☒	☐	MRI-based neuroimaging

Human research participants

Policy information about studies involving human research participants

Population characteristics

We included adult patients (18+) that were admitted at least once to the operating theatre for cardiothoracic surgery at Deutsches Herzzentrum Berlin, Germany, between October 2012 and February 2018. The patient characteristics were well balanced between the training and the test set. Median age was 72 years in the training set and 70 years in the test set. 64% (training set) and 67% (test set) were male in the study population. Please see Supplementary Tables 1-4, 8 and 10 for further description of the patient characteristics.

Recruitment

The data was recruited from the electronic health record system of Deutsches Herzzentrum Berlin, Germany. Patients without any creatinine or urine flow values, patients receiving hemodialysis before the end of the operation or having a baseline

creatinine level >= 4.0mg/dl were excluded. Additionally, 28 patients were excluded from the 392 patients of the set eligible for validation due to insufficient documentation of the type of surgery, false recording of surgery times or notion of end-stage kidney disease in the patients' history that was not detected by automated filtering.

Ethics oversight	The study was approved by the institutional data protection officer and ethics committee (EA2/180/17). The approval included the collection of data on implied consent. We only used retrospective data and the patients were not actively involved in the study. The requirement of informed consent of the participating physicians was waived by the Institutional Review Board due to anonymized data acquisition.

Note that full information on the approval of the study protocol must also be provided in the manuscript.
